# Histopathological pulmonary findings of survivors and autopsy COVID-19 cases: A bi-center study

**DOI:** 10.1097/MD.0000000000032002

**Published:** 2022-11-25

**Authors:** Magdalena Chrabańska, Agnieszka Mazur, Katarzyna Stęplewska

**Affiliations:** a Department and Chair of Pathomorphology, Faculty of Medical Sciences in Zabrze, Medical University of Silesia, Katowice, Poland; b Department of Ophthalmology, Regional Specialised Hospital No. 4, Bytom, Poland; c Department of Pathology, Institute of Medical Sciences, University of Opole, Opole, Poland.

**Keywords:** autopsy, COVID-19, lung, pneumo-hematocele, SARS-CoV-2

## Abstract

The coronavirus disease 2019 (COVID-19), caused by Severe Acute Respiratory Syndrome Coronavirus-2 (SARS-CoV-2), evolved into a global pandemic. As ACE2 on the surface of alveolar cells of the lung epithelium is one of the potential target receptors for SARS-CoV-2, the respiratory symptoms are the most common presentation of COVID-19. The aim of our study was to investigate the morphological findings in lung tissue after being infected by SARS-CoV-2 and compare histopathologic changes in patients with COVID-19 infection history who died to those who survived. We analyzed lung tissue samples from 9 patients who died from COVID-19 and from 35 patients with COVID-19 infection history who survived and had undergone lung surgery for different reasons. Most of histopathological changes in autopsy and survivors’ cases overlapped; however, they occurred with different frequency. The predominant histologic finding both in the case of the deceased and the survivors was patchy distribution of foamy macrophages in the alveolar spaces. In comparison with autopsy cases viral cytopathic-like changes in hyperplastic pneumocytes were rarely observed in the survivors’ lung tissue. Pulmonary edema, fibrin deposition within alveoli, bronchopneumonia, small vessel thrombosis and type II pneumocyte hyperplasia were also more often observed within autopsy cases. Life-threatening complications such as hyaline membrane formations and diffuse alveolar damage were present only within the deceased, whereas changes requiring enough time to progress to the fibrotic phase, such as organizing pneumonia, bronchiolization of the alveoli, and interstitial fibrosis were observed in the lung parenchyma only in survivors. Additionally, 14 cases of pulmonary pneumo-hematocele in patients with COVID-19 infection history who survived were observed. It is a newly observed entity in the form of a cystic lesion formed by large accumulation of blood and fibrin between the collapsed and rejected lung parenchyma and/or present with air–fluid levels. The thin wall of pneumo-hematocele is formed by the inter lobar interstitial fibroconnective tissue and has no epithelial lining or bronchial wall elements. As the COVID-19 pandemic continues, new complications following SARS-CoV-2 infection are identified. Newly observed entity in patients with COVID-19 infection history who survived is pulmonary pneumo-hematocele. The appearance of these lesion has become increasingly frequent.

## 1. Introduction

The coronavirus disease 2019 (COVID-19), caused by Severe Acute Respiratory Syndrome Coronavirus-2 (SARS-CoV-2), first appeared in December 2019, in Wuhan, China and evolved into a global pandemic.^[1]^ As angiotensin-converting enzyme 2 (ACE2) is one of the potential target receptors for SARS-CoV-2 in human body, which is usually present on the surface of alveolar cells of the lung epithelium, the respiratory symptoms are the most common presentation of COVID-19.^[2,3]^ Proceeded pulmonary injury with massive alveolar damage and progressive failure leads to severe disease progression and can result in death.^[3]^ The risk of death is greater in men than in women, increases with age, and is elevated in patients with the number of coexisting diseases, mostly with chronic cardiovascular, respiratory, and metabolic diseases.^[3,4]^ On the other hand, patients who survived are at risk of developing various complications, such as cardiac disorders,^[5,6]^ neurologic affection,^[7,8]^ and pulmonary fibrosis.^[9,10]^ Recently, the appearance of characteristic pulmonary lesions such as pneumo-hematocele or giant bulla has been noted.^[11–16]^ There have been published many papers documenting postmortem pathology and discussing pathophysiology in COVID-19.^[1,3,17–36]^ However, the number of research analyzing the histopathologic changes in the lungs of patients with COVID-19 infection history who survived and developed pulmonary complications is still insufficient.

Hence, the aim of this study was to investigate the morphological findings in the lung tissue from patients with COVID-19 infection history who died and those who survived. Moreover, we described histopathologic features of recently known post—COVID-19 pulmonary complication in the form of pneumo-hematocele.

## 2. Methods

From October 2020 to July 2021, a retrospective study was performed in patients admitted with a diagnosis of SARS-CoV-2 infection in 2 hospitals. The Institutional Review Board of Medical University of Silesia, Katowice, Poland waived the need for ethical approval for this research (PCN/CBN/0022/KB/225/21). The study was performed in accordance with the Declaration of Helsinki and patient data were kept fully anonymous in all steps.

Lung tissue samples from 35 patients with COVID-19 infection history who survived and had undergone lung surgery between November 2020 and July 2021 and from 9 patients who died from COVID-19 between October 2020 and August 2021 were systematically analyzed. All patients had SARS-CoV-2 infection confirmed by real-time polymerase chain reaction analysis of throat swab samples.

All 35 patients underwent surgery at Clinic of Thoracic Surgery, Faculty of Medical Sciences in Zabrze, Medical University of Silesia, Katowice, Poland. Autopsies were done in Chair of Pathomorphology, Faculty of Medical Sciences in Zabrze, Medical University of Silesia, Katowice, Poland (3 autopsies) and in Department of Pathomorphology, University Clinical Hospital in Opole, Poland (6 autopsies).

The submitted surgical lung segmental or lobectomy specimens and postmortem lung specimens were handled according to the current guidelines of the Polish Society of Pathologists.

A median of 6 tissue blocks (range 4–8) were taken from lung tissue, selecting the most representative areas identified at macroscopic examination. All tissue specimens were formalin-fixed for at least 24 h. Paraffin-embedded sections of 4-μm thickness were stained with hematoxylin and eosin.

Histological evaluation of all sections from all cases was done by 2 independent pathologists from each hospital, who were blinded to patients’ clinicopathological details. Each pathologist analyzed all the slides from both hospitals. Inconsistency in the results were discussed to reach a consensus for all samples and in order to avoid potential sources of bias. Quantitative data was summarized into frequency tables.

In reporting this study, the standard methodology was reported according to “Strengthening the Reporting of Observational Studies in Epidemiology” (STROBE) guidelines.

## 3. Results

### 3.1. Autopsy cases

The patients were 4 women and 5 men, with a mean age of 71 years (range 58–85). All patients died within 30 days of symptom onset of disease. Regarding past comorbidities, data were available for all patients: 8 patients (88.9%) had cardiovascular disorders, 3 patients (33.3%) were obese, 2 patients (22.2%) had hypertension, and one patient (11.1%) had past malignancies. None of the patients had diabetes or chronic obstructive pulmonary disorders.

Upon gross examination, the lungs of all patients were enlarged, heavy, congested, and edematous with patchy involvement. The parenchyma was airless and had areas ranging from pale pink or gray to dark red in color with scattered ill-defined hemorrhagic areas. Macroscopic thrombi were evaluable in 3 cases (33.3%)–in the pulmonary trunk (one case) and in small vessels (2 cases). The trachea was erythematous and dull in 2 cases (22.2%). The bronchi were filled with mucopurulent or hemorrhagic fluid in 4 patients (44.4%). The hilar lymph nodes were slightly enlarged (up to 3 cm) in 4 cases (44.4%). Accumulation of serous fluid in pleural cavities (up to 300 mL in every cavity) were present in 2 patients (22.2%).

**Figure 1.**
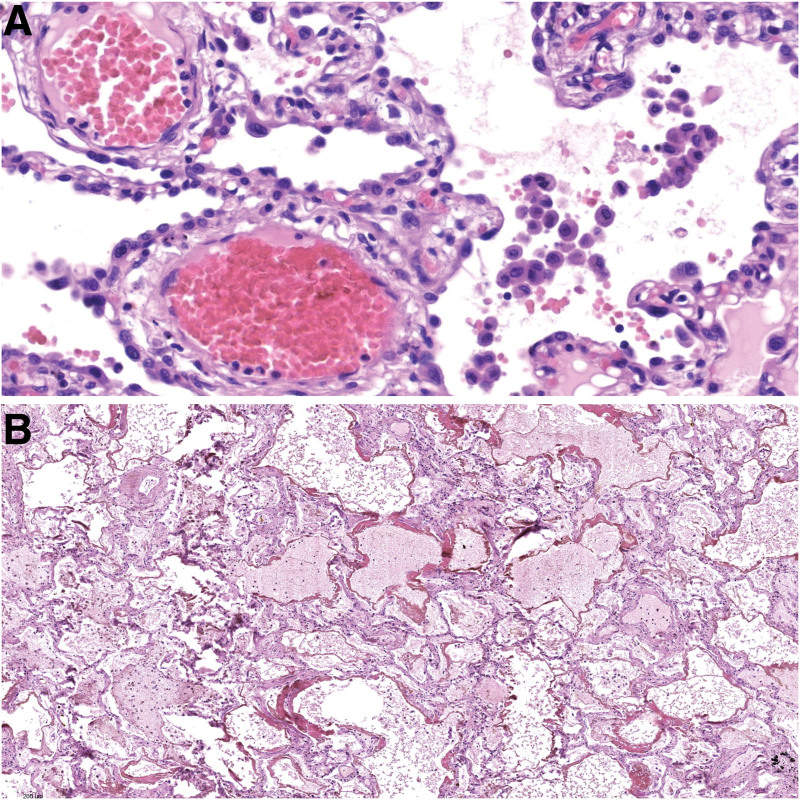
Frequent histological findings in autopsy cases: (A) alveolar macrophages in the alveolar spaces and capillary congestion (HE, 285×); (B) hyaline membranes formation (HE, 60×).

**Figure 2.**
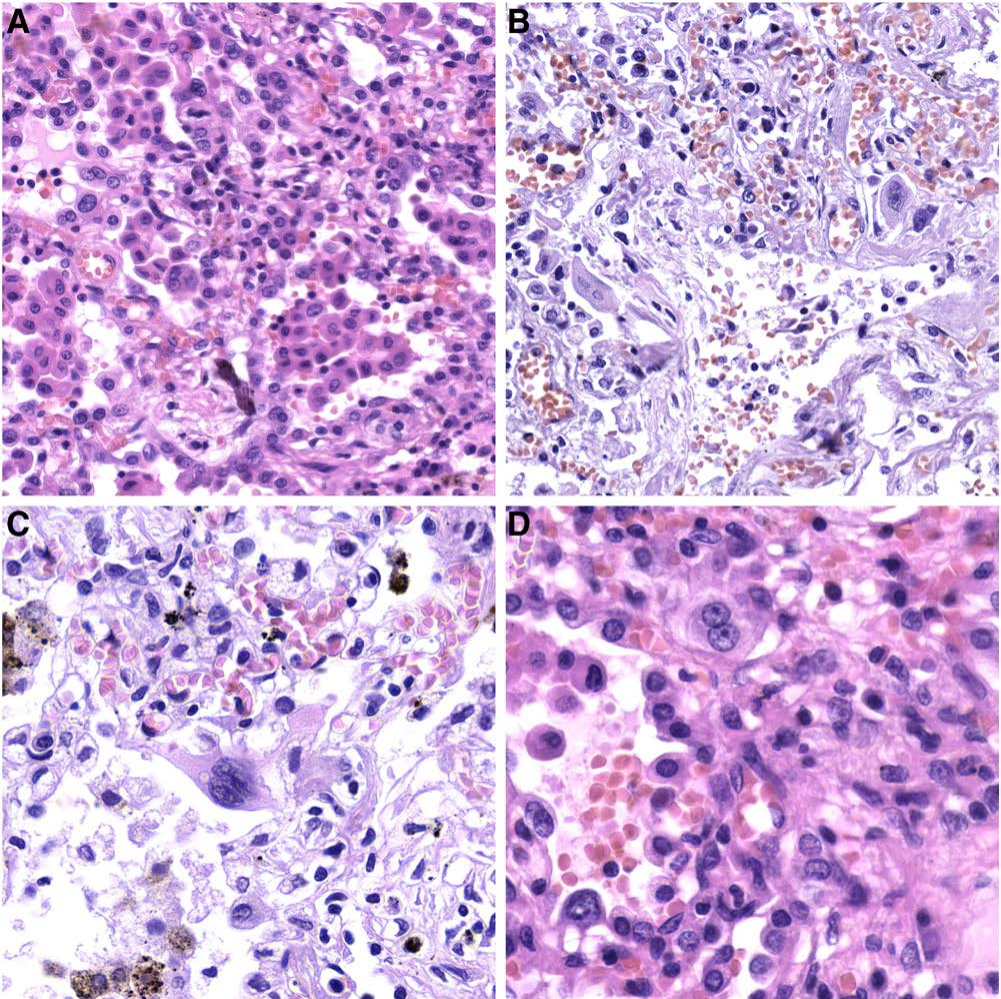
A panel of microphotographs of the reactive changes in hyperplastic pneumocytes: (A) cellular atypia (HE, 345×), (B) enlarged nuclei (HE, 341×), (C) multinucleation (HE, 549×), (D) binucleated cells with prominent nucleoli (HE, 700×).

In all of the cases, histological examination revealed alveolar macrophages in the alveolar spaces (Fig. 1A). Capillary congestion (Fig. 1A), formation of the hyaline membranes composed of serum proteins and condensed fibrin (Fig. 1B), features corresponding to the acute phases of diffuse alveolar damage (DAD), emphysema, and reactive changes in the hyperplastic pneumocytes were also observed in almost all of the patients (77.8%–88.9%). Reactive changes in hyperplastic pneumocytes included various aspects of binucleated cells, cellular atypia, enlarged nuclei, amphophilic granular cytoplasm, and prominent nucleoli (Fig. 2). These reactive changes in the hyperplastic pneumocytes were reminiscent of viral cytopathic-like changes; however, obvious intranuclear or intracytoplasmic viral inclusions were not detected. Patchy mild increase of interstitial inflammatory lymphomonocytic infiltrate along the slightly thickened interalveolar septa were also often noted (66.7%). Microscopic autopsy findings such as atelectasis, intra-alveolar fibrin deposition, intra-alveolar edema, acute bronchopneumonia, and small vessel thrombosis with alveolar hemorrhage were observed in about half of the patients (44.4%–55.6%). Features corresponding to the organizing phase of DAD were present in only 1 patient (11.1%). There were no areas of organizing pneumonia with fibroblastic proliferations or Masson bodies within the alveolar ducts or bronchioles, and no features of the fibrotic phase of DAD, such as marked parenchymal fibrosis, micro-honeycombing, or architectural remodeling. This indicates that none of the patients had progressed to the fibrotic phase, possibly because of the too short duration of the infection. Histological examination of the main bronchi and bronchiolar branches revealed focal squamous metaplasia, while their lumina contained mucoid material in a few cases (22.2%). Moreover, type II pneumocyte hyperplasia and neutrophils clotting within small capillaries around alveoli were found among microscopic findings in a subset of patients (22.2%–33.3%). The microscopic findings observed within autopsy cases were summarized in Table 1.

**Table 1:** The frequency of microscopic findings observed within autopsy and survivors cases.

Pulmonary pathology	Autopsy cases (%)	Survivors cases (%)
Alveolar macrophages	100.0	83.9
Capillary congestion	88.9	64.5
Reactive changes in hyperplastic pneumocytes	77.8	3.2
Emphysema	77.8	64.5
Lymphocytic inflammation	66.7	48.4
Atelectasis	55.6	64.5
Intra-alveolar fibrin deposition	55.6	19.4
Oedema	55.6	29.0
Acute bronchopneumonia	55.6	22.6
Alveolar hemorrhage	44.4	64.5
Small vessel thrombosis	44.4	9.7
Type II pneumocyte hyperplasia	33.3	58.1
Mucus in airways	22.2	19.4
Bronchial squamous metaplasia	22.2	12.9
Neutrophils clotting	22.2	22.2
Hyaline membrane	88.9	0
DAD–acute	88.9	0
DAD–organizing	11.1	0
Pulmonary hematocele	0	40.0
Organizing pneumonia	0	32.3
Bronchitis	0	25.8
Hemorrhagic infarct	0	16.1
Bronchiolization of the alveoli	0	16.1
Interstitial fibrosis	0	12.9
Thickening of alveolar capillaries	0	3.2

DAD—diffuse alveolar damage.

### 3.2. Survivors’ cases

The patients were 4 women and 31 men, with a mean age of 53.3 years (range 32–84). The mean time from symptoms onset to the thoracosurgical intervention was 3 months (range 1–5). Regarding comorbidities data were available for all of the patients: 8 patient (25.8%) had past malignancies (lung neoplasms), 8 patients (25.8%) had cardiovascular disorders, 5 patients (16.1%) had hypertension, and 1 patient (3.2%) had type 2 diabetes. With regards to the purposes of the lung surgery: pneumothorax (n = 11, 31.4%), lung malignant neoplasm (n = 8, 25.8%), pulmonary hematocele (n = 7, 20.0%), abscess (n = 5, 16.1%), pleural empyema (n = 4, 11.4%).

**Figure 3.**
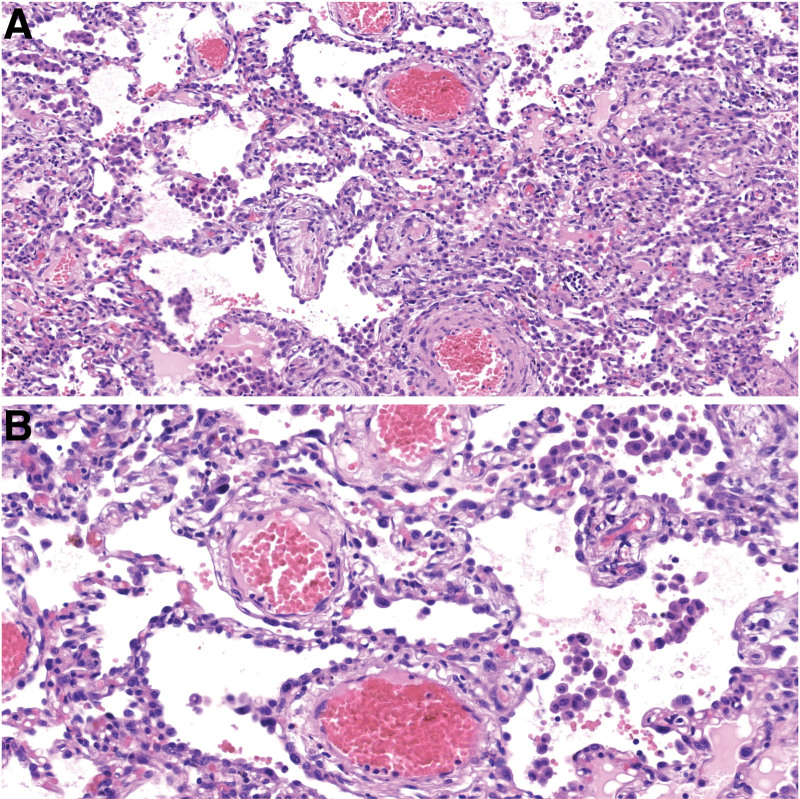
Type II pneumocyte hyperplasia (A: HE, 150×, B: HE, 285×).

In the majority of patients (64.5%–83.9%) the prominent histologic findings were patchy distribution of intra-alveolar foamy macrophages filling some airspace, capillary congestion, emphysema, alveolar hemorrhage and atelectasis. Mild infiltration of lymphocytes in the interstitial regions and type II pneumocyte hyperplasia (Fig. 3) were also often noted during microscopic examination (48.4%–58.1%).

**Figure 4.**
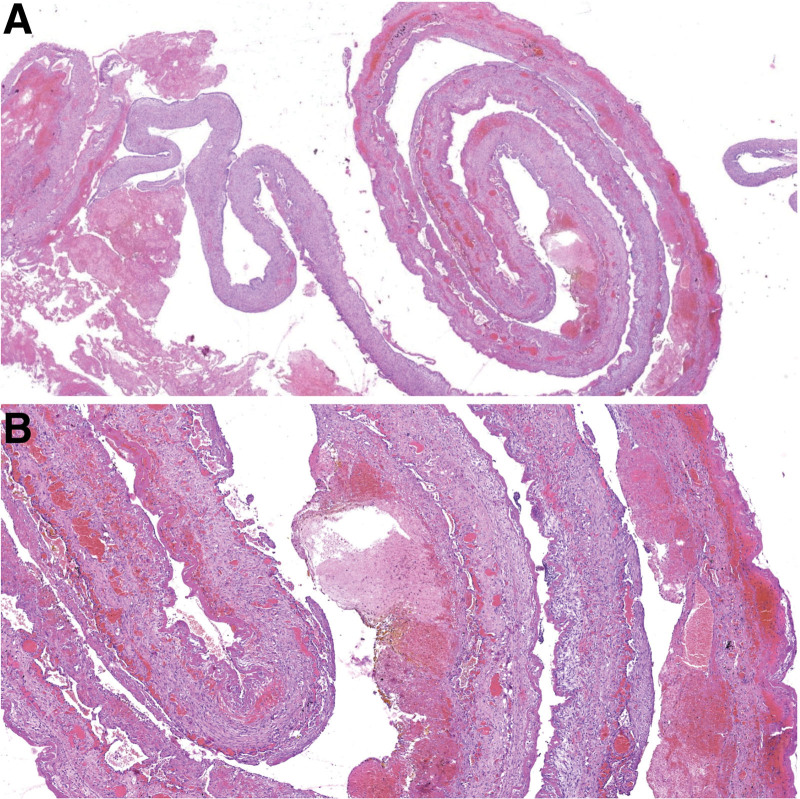
Pulmonary hematocele (A: HE, 8×, B: HE, 34×).

A very interesting and relatively common (observed in 14 out of 35 patients) finding was pulmonary pneumo-hematocele. It is a cystic lesion formed by large accumulation of blood and fibrin between the collapsed and rejected lung parenchyma and/or present with air–fluid levels. The thin wall of pneumo-hematocele is formed by the inter lobar interstitial fibroconnective tissue and has no epithelial lining or bronchial wall elements (Fig. 4). The microscopic findings observed in the lung parenchyma surrounding the pneumo-hematocele were summarized in Table 2. All patients with pulmonary pneumo-hematocele were men, with a mean age of 47.8 years (range 35–72). Of these patients, 7 had pre-operatively diagnosed pseudocyts. The other reasons for which the patients with pulmonary hematocele underwent surgery were pneumothorax (n = 6, 42.9%) and pleural empyema (n = 2, 14.3%).

**Table 2:** The frequency of microscopic findings observed in patients with pulmonary hematocele.

Pulmonary pathology	Cases (%)
Alveolar macrophages	57.1
Atelectasis	50.0
Capillary congestion	42.9
Alveolar hemorrhage	42.9
Emphysema	35.7
Organizing pneumonia	35.7
Oedema	28.6
Lymphocytic inflammation	28.6
Type II pneumocyte hyperplasia	21.4
Bronchial squamous metaplasia	14.3
Reactive changes in hyperplastic pneumocytes	14.3
Mucus in airways	7.1
Intra-alveolar fibrin deposition	7.1
Bronchitis	7.1

Acute bronchopneumonia with abscess formation, small vessel thrombosis, neutrophils clotting, hyaline membrane formation, DAD—acute and organizing phases, hemorrhagic infarct, bronchiolization of the alveoli, interstitial fibrosis, and thickening of alveolar capillaries have not been recorded in any patient with pulmonary pseudocyst.

**Figure 5.**
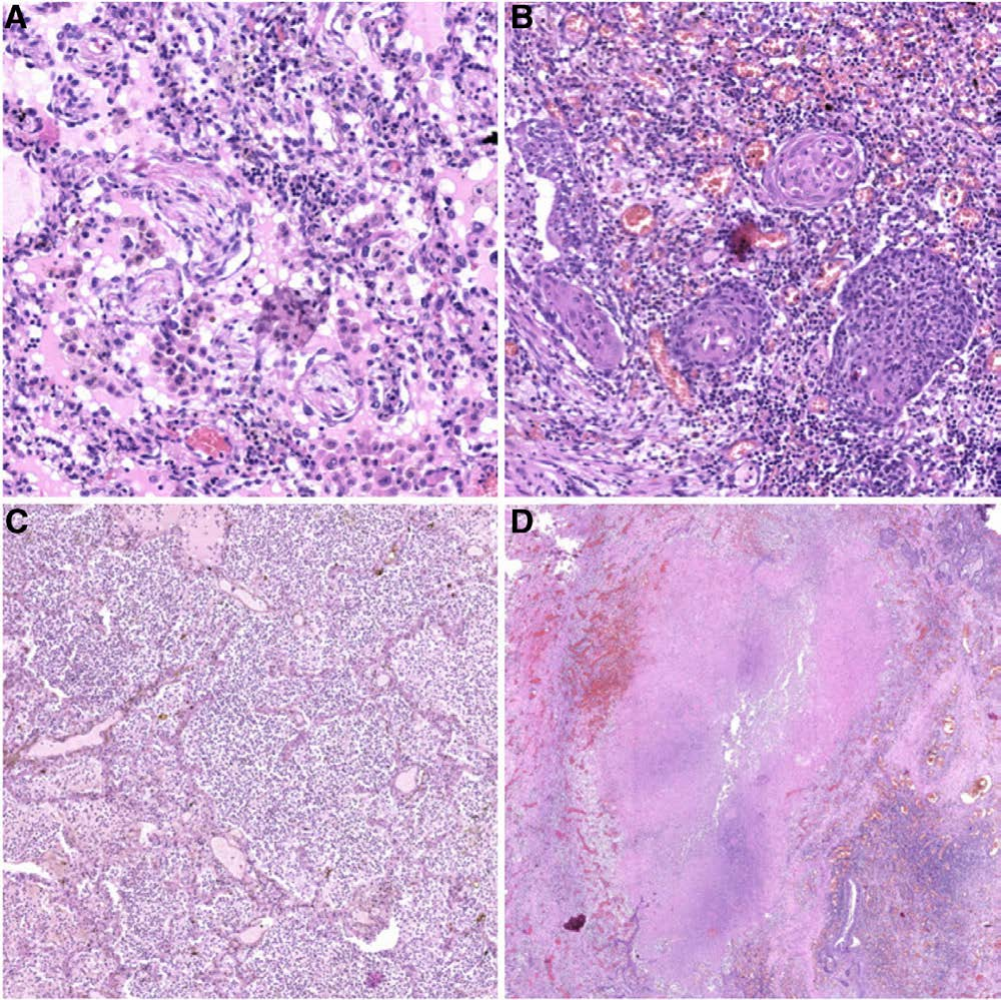
A panel of microphotographs of common microscopic findings in survivors cases: (A) organizing pneumonia with alveolar loose plugs of fibroblastic tissue (HE, 185×), (B) squamous metaplasia of bronchial epithelium (HE, 150×), (C) acute bronchopneumonia (HE, 60×), (D) abscesses formation (HE, 15×).

About one-third of the survivors’ cases (32.3%) were characterized by organizing pneumonia with alveolar loose plugs of fibroblastic tissue (Fig. 5A). Furthermore, the bronchi revealed focal squamous metaplasia (Fig. 5B) and mild lymphocytic and monocytic infiltrate, and their lumina contained residual dense mucoid material in a subset of patients (12.9%–48.4%). Other findings included intra-alveolar edema and condensed fibrin deposition within alveoli. Focal or diffuse bronchopneumonia (Fig. 5C) with abscesses formation (Fig. 5D) has been not uncommonly (22.6%) observed as a result of bacterial superinfection. Interestingly, although bronchopneumonia can be associated with existing mass and post-obstructive-type changes, the neoplasm was observed in only 1 in 7 cases with bronchopneumonia. Hemorrhagic infarct, bronchiolization of the alveoli and interstitial fibrosis were relatively frequently observed (16.1%) microscopic findings. In all cases with hemorrhagic infarct the accompanying finding was capillary congestion, while thromboemboli in the lumina of small vessels were identified only in 1 patient with hemorrhagic infarct. Rare phenomena (9.7%–22.2%) in the group of survivors’ cases included thrombi and neutrophils clotting within small vessels. Both desquamated type 2 pneumocytes within the alveolar spaces with cytomegaly and enlarged nuclei with bright eosinophilic nucleoli and thickening of the alveolar capillaries were observed only in the individual cases (3.2%). The microscopic findings observed within survivors’ cases were summarized in Table 1.

## 4. Discussion

Since the first postmortem pathology in a decedent with SARS-CoV-2 was reported in February 2020,^[37]^ many papers documenting postmortem findings and discussing pathophysiology in COVID-19 have been published.

Microscopic autopsy findings have been reported with a great variability. Regarding autopsy cases, all results are generally consistent with previous research. Consistent with current literature, the most frequently demonstrated pulmonary pathology is mainly acute or less often organizing phase of DAD, or both injury patterns together.^[1,3,20,22,24–26,28,30,31,38–41]^ Both of these phases of DAD were also observed in this study; however, they were not a predominant histologic feature among autopsy cases and the frequency of them were lower in comparison with cited research. Infiltration of lymphocytes in the interstitial regions, type II pneumocyte hyperplasia (characterized by atypically enlarged pneumocytes with large nuclei, amphophilic granular cytoplasm, and prominent nucleoli), intraalveolar fibrin deposition, and formation of hyaline membranes composed of serum proteins and condensed fibrin were the most commonly observed histopathological changes after DAD described by most studies.^[3,33,37,38,42,43]^ The same microscopic findings in the lung tissue of the deceased were also observed in our study. In addition, small vessels thrombosis and fresh thrombi in the pulmonary arteries could be detected in many COVID-19 patients.^[2,3,22,38–40]^ This phenomenon represents a histological correlate of coagulopathies occurring in this infection. Desquamated type II pneumocytes within the alveolar spaces showing viral cytopathic changes were found among microscopic findings in different postmortem studies.^[3,28,39]^ Cytomegaly and enlarged nuclei with bright eosinophilic nucleoli which are reminiscent to the described in the literature cytopathic effects were also observed in our research; however, we favor that they are in the spectrum of reactive changes. As in other studies, obvious intranuclear or intracytoplasmic viral inclusions were not detect in our study.^[3,39,40]^ Other prominent lung pathological conditions that have been described and proved by observations in our study include severe capillary congestion, alveolar hemorrhage, intra-alveolar edema, collapsed alveoli and patchy distribution of intra-alveolar foamy macrophages filling some airspace throughout all lobes.^[2,3,20,38,40]^ Focal or diffuse bronchopneumonia with possible abscess formation has been also observed in some of the deceased.^[1,38,40]^ It is not yet clear whether pneumonia represents an aspect of the reaction to viral infection or bacterial superinfection in the majority of cases. However, most pathologists attribute acute pneumonia to superinfection which is common in viral pneumonias, and we also subscribe to that opinion. Histological examination of the main bronchi and bronchiolar branches can reveal in some cases focal squamous metaplasia and mild transmural lymphocytic infiltrate, while their lumina often contain residual dense mucoid material and granulocytes.^[2,38,44]^ These observations are consistent with ours; however, not all researchers noticed obvious signs of long-term processes such as squamous metaplasia.^[3,19]^ Additional microscopic findings described in the literature but not observed in our research include thickening of the alveolar capillaries, cytoplasmic vacuolization in the pulmonary arteries, increased number of megakaryocyte with multinuclear appearance within the branching small vessels and thrombotic microangiopathy which is consistent with complement-mediated microvascular injury.^[2,3,20,24,26,39,40]^ Areas of organizing pneumonia with fibroblastic proliferations or Masson bodies and features indicative of the fibrotic phase of DAD, such as architectural remodeling, mural fibrosis, and microcystic honeycombing were not observed or at most were focal both in our and other studies.^[3,38]^ It suggests that none of the patients had progressed to the fibrotic phase, possibly because of the too short duration of the disease.

Although many papers documenting postmortem findings in COVID-19 have been published, there is less research reporting on the morphological features of the lung tissue in the survivors of COVID-19 infection. Most of the histopathological changes in the autopsy and survivors’ cases overlap; however, they occur with different frequency. Life-threatening complications such as hyaline membrane formations and DAD—acute and organizing phases—were not present within survivors, whereas changes requiring enough time to progress to the fibrotic phase, such as organizing pneumonia, bronchiolization of the alveoli, and interstitial fibrosis were observed in the lung parenchyma only among survivors.

Pulmonary pneumo-hematocele is one of the most characteristic long-term respiratory sequelae of COVID-19 infection reported so far only in several cases.^[11–16]^ A pneumatocele is an air-filled pseudocyst, with a thin wall that forms in the lung interstitium. If it is filled with blood, it is called pneumo-hematocele.^[11,15,45]^ Some authors report similar findings and describe the airspace as a bulla rather than a pneumatocele.^[46]^ Most pneumo-hematoceles occur in relatively young male patients (most often in the age of 30–40) and generally in those with the moderate and severe disease around day 20 after diagnosis. Pneumo-hematoceles can present with symptoms due to pneumothorax, lung displacement, and rupture or as asymptomatic lesion in imaging studies.^[11]^ The precise pathogenesis of pneumo-hematatocele remains uncertain. Most authors agree with the theory that after infectious pneumonia the inflammatory and airway secretions form a bronchial valve, which allows the air to enter but not to come out, this results in a rupture of the airways and formation of air-filled cysts.^[11,14–16,45]^ Another theory suggests that inflammation and necrosis of a portion of the airway results in focal collections of air in the interstitial tissue and formation of grossly identifiable pneumatocele.^[16,47]^ Coronavirus targets alveolar epithelial cells, which together with cytokine storm make the alveoli vulnerable to rupture with subsequent air leakage.^[16,48]^ Air leaked in the interstitium causes injury to small blood vessels, which can explain the presence of blood clots and bloody fluid inside the cyst.^[16]^ The majority of pneumatoceles usually resolve spontaneously within few weeks of onset; however, it may rapidly increase in size especially in patients on mechanical ventilation or undergoing general anesthesia.^[16]^ Moreover, in some instance the pneumatocele can dissect through the pleural membrane causing pneumothorax.^[11,14,15,49]^

Our study has some limitations that must be considered. The primary limitation to the generalization of our results is the relatively small number of cases included in this study due to a fairly short time since the onset of the pandemic, which does not allow long follow-up of patients. The subsequent limitation concerns the limited number of prior research on this topic. Most of the available studies concern postmortem pathology and pathophysiology in COVID-19, while the number of research analyzing the histopathologic changes in the lungs of patients who survived and developed pulmonary complications is still insufficient.

## 5. Conclusion

As the COVID-19 pandemic continues, new complications following SARS-CoV-2 infection are identified. Most of the histopathological changes in autopsy and survivors’ cases overlapped; however, they occurred with different frequency. The predominant histologic finding both in the case of the deceased and the survivors was patchy distribution of foamy macrophages in the alveolar spaces. In comparison with autopsy cases viral cytopathic-like changes in hyperplastic pneumocytes were rarely observed in the survivors’ lung tissue. Life-threatening complications such as hyaline membrane formations and DAD were present only within the deceased, whereas changes requiring enough time to progress to the fibrotic phase, such as organizing pneumonia, bronchiolization of the alveoli, and interstitial fibrosis were observed in the lung parenchyma only in survivors. Additionally, a newly observed entity in patients with COVID-19 infection history who survived was pulmonary pneumo-hematocele—a cystic lesion formed by large accumulation of blood and fibrin between the collapsed and rejected lung parenchyma and/or present with air-fluid levels. Its appearance has become increasingly frequent; however, it still requires further observations and confirmation in subsequent studies.

## Author contributions

**Conceptualization:** Magdalena Chrabańska, Agnieszka Mazur, Katarzyna Stęplewska.

**Data curation:** Magdalena Chrabańska, Agnieszka Mazur.

**Formal analysis:** Magdalena Chrabańska, Agnieszka Mazur, Katarzyna Stęplewska.

**Funding acquisition:** Magdalena Chrabańska.

**Investigation:** Magdalena Chrabańska, Katarzyna Stęplewska.

**Methodology:** Katarzyna Stęplewska.

**Resources:** Magdalena Chrabańska.

**Visualization:** Magdalena Chrabańska, Agnieszka Mazur.

**Writing—original draft:** Magdalena Chrabańska

**Writing—review and editing:** Katarzyna Stęplewska.
